# Feasibility of Screening Primary Aldosteronism by Aldosterone-to-Direct Renin Concentration Ratio Derived from Chemiluminescent Immunoassay Measurement: Diagnostic Accuracy and Cutoff Value

**DOI:** 10.1155/2019/2195796

**Published:** 2019-07-02

**Authors:** Tianqi Li, Yeshuo Ma, Ying Zhang, Yue Liu, Tingting Fu, Ri Zhang, Kai Kang, Yingchao Yang, Lixin Wang, Yinong Jiang, Yan Lu

**Affiliations:** ^1^Department of Cardiology, Institute of Cardiovascular Diseases, First Affiliated Hospital of Dalian Medical University, Dalian, Liaoning, China; ^2^Xiangya School of Pharmaceutical Sciences, Central South University, Changsha, Hunan, China; ^3^Department of Cardiology, Dalian Ganjingzi District People Hospital, Dalian, Liaoning, China; ^4^Cardiac Echocardiography, First Affiliated Hospital of Dalian Medical University, Dalian, Liaoning, China

## Abstract

**Objectives:**

Aldosterone-to-plasma renin activity ratio (ARR) derived from traditional radioimmunoassay (RIA) is widely used to detect primary aldosteronism (PA). Recently, aldosterone-to-direct renin concentration ratio (ADRR), which is calculated by direct renin concentration (DRC) measured by chemiluminescent immunoassay (CLIA), is proposed to replace ARR as the screening test method for PA. The purpose of the present study was to estimate the diagnostic accuracy and cutoff value of ADRR as screening test for PA.

**Methods:**

450 hypertensive patients with suspected PA referred to hypertension center of our department were enrolled and underwent screening and confirmatory tests of PA. Plasma renin activity (PRA), DRC, and plasma aldosterone concentration (PAC) were measured by both RIA and CLIA simultaneously during screening and confirmatory test.

**Results:**

386 patients were diagnosed as primary hypertension (PH) and 64 patients as PA. Within-patient correlation between PRA and DRC (r=0.88, P<0.001) and correlation between PAC measured by RIA and CLIA were high (r=0.80, P<0.001). The optimal cutoff value of ADRR was 2.93 (ng/dL)/(mU/L), sensitivity 80.33%, and specificity 92.11%. The optimal cutoff value of ARR was 25.28 (ng/dL)/(ng/mL/h), sensitivity 76.92%, and specificity 93.38%.

**Conclusion:**

The optimal cutoff values of ADRR and ARR for screening PA are defined in this patient cohort with high sensitivity and specificity. Our results are of clinical importance for accelerating the extensive use of ADRR as a screening test for PA in daily practice.

## 1. Introduction

Primary aldosteronism (PA) is characterized by excessive and autonomous aldosterone production and suppressed plasma renin and is commonly caused by aldosterone-producing adenoma (30%-50%) and bilateral adrenal hyperplasia (50%-65%) [1, 2]. PA is a curable and the most common form of secondary hypertension [3]. The prevalence of PA is estimated around 5% to 10% among hypertensive patients [4, 5]. It is known that patients with PA are associated with significant higher risk of cardiovascular events and target organ damage than patients with primary hypertension (PH) at comparable blood pressure level [4, 6]. Since the treatment of patients with PA is different from that of patients with PH, early diagnosis of PA is of clinical importance, which is essential for appropriate targeted management leading to prognosis improvement. Up to 50% of hypertensive patients should undergo screening for PA, including hypertensive patients with grade 2-3 and resistant hypertension and hypertensive patients with hypokalemia independent of blood pressure levels [4, 7].

A reliable and convenient screening test is essential for diagnosis of PA [4]. Traditionally, aldosterone-to-plasma renin activity ratio (ARR) is considered to be the choice of screening test [4, 8]; plasma aldosterone concentration (PAC) and plasma renin activity (PRA) are derived from traditional radioimmunoassay (RIA). PRA is indirectly estimated by efficiency of generating angiotensin I from angiotensinogen, which might be affected by the concentration of angiotensinogen in plasma [9]. In addition, this method is time consuming and produces radioactive waste. Nowadays, aldosterone-to-direct renin concentration ratio (ADRR) is under development and proposed to replace ARR as the screening test for PA; ADRR is known to have high accuracy and reproduction efficacy in detecting patients with PA [10–12]. ADRR is calculated by direct renin concentration (DRC) measured by chemiluminescent immunoassay (CLIA) on automated platform; this method is reproducible, simpler, and less time consuming as compared to ARR method. Previous researches verified the relationship between DRC measured by CLIA and PRA measured by RIA [13, 14]. However, ADRR has not been widely used in routine diagnosis of PA, because more evidence is needed to define the accurate cutoff value and diagnostic efficiency of ADRR for screening PA.

The aim of this study was to investigate the diagnostic accuracy and optimal cutoff value of ADRR as screening test for PA in referred hypertensive patients based on a proper sample capacity.

## 2. Materials and Methods

### 2.1. Patients Selection

**Figure 1:**
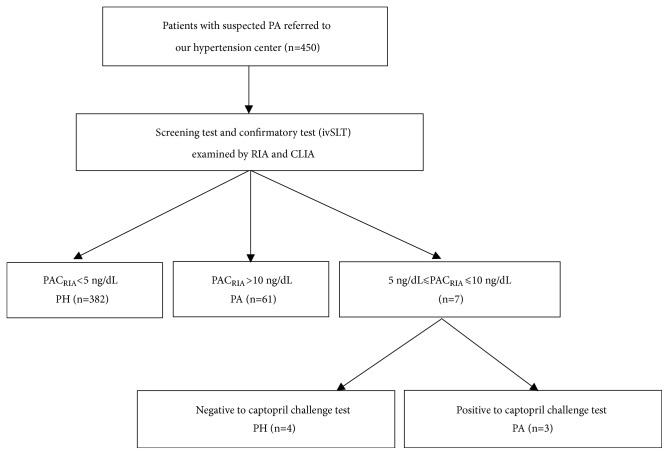
*Flowchart of the study.* 450 hypertensive patients with suspected PA were enrolled. After screening and confirmatory tests, 386 patients were diagnosed with PH and 64 patients with PA. PH: primary hypertension; PA: primary aldosteronism; RIA: radioimmunoassay; CLIA: chemiluminescent immunoassay; PAC_RIA_: plasma aldosterone concentration measured by radioimmunoassay; ivSLT: intravenous saline loading test.

In this retrospective study, we collected clinical data of patients hospitalized with suspected PA in the hypertension center of our department between March 2016 and July 2018. Based on previous experience [13], 440 patients (44 PA patients) were needed to achieve acceptable results. Pregnant women and patients with heart failure, liver and kidney dysfunction, malignant tumor, malnutrition, and any other major illness that could affect life expectancy and/or renin-angiotensin-aldosterone system were excluded. The clinical characteristics of all patients, including age, sex, body mass index (BMI), systolic blood pressure (SBP), diastolic blood pressure (DBP), hear rate (HR), serum K^+^/Na^+^, urinary K^+^/Na^+^, and serum creatinine were summarized in Table 1. All patients underwent the following prescreening preparation examinations before performing screening test: pharmacological wash-out, regulation of serum potassium and dietary modification. Aldosterone receptor antagonists were withdrawn for at least 6 weeks; diuretic was withdrawn for at least 4 weeks; angiotensin converting enzyme inhibitors, angiotensin II receptor blockers, *β*-blockers, dihydropyridine calcium blockers, and clonidine were withdrawn for at least 2 weeks. Patients were prescribed non-dihydropyridine calcium blocker (diltiazem) and/or *α*-blockers (doxazosin, terazosin) to control blood pressure. The flowchart of the present study is shown in Figure 1. The screening test and confirmatory test (intravenous saline loading test, ivSLT) were performed and determined according to the Endocrine Society guidelines [4]. PA was diagnosed by RIA at confirmatory test. In the present study, PRA, DRC, and PAC were determined by both RIA and CLIA simultaneously during screening and confirmatory tests. The detection ranges and sensitivities of them were shown in Table 2. For confirmatory test, if PAC measured by RIA was between 5 and 10 ng/dL, additional captopril challenge test was performed. This study was approved by the Ethics Committee of First Affiliated Hospital of Dalian Medical University. Written informed consent was obtained from all the patients.

### 2.2. Biochemical Measurements

Blood samples were centrifuged and tested immediately after collection. PRA and PAC_RIA_ were measured by RIA with the RENCTK RIA kit (DiaSorin, Saluggia, Italy) and ALDOCTK-2 (DiaSorin, Saluggia, Italy) according to the manufacturer's instructions. DRC and PAC_CLIA_ were detected by CLIA using the LIAISON® Direct Renin kit (DiaSorin, Saluggia, Italy) and the LIAISON® XL Aldosterone kit on the corresponding fully automated analyzer (DiaSorin, Saluggia, Italy) according to the manufacturer's protocol. The equations of ARR and ADRR are as follows: ARR= PRA/PAC_RIA_; ADRR=DRC/  PAC_CLIA_.

### 2.3. Statistical Analysis

Statistical analysis was performed using SPSS software (version 20, IBM Corporation, Armonk, NY, USA). Normally distributed variables were expressed as mean ± SD, and non-normally distributed variables were expressed as median (25th to 75th percentile). Since PRA, DRC, PAC_RIA_ (PAC measured by RIA), and PAC_CLIA_ (PAC measured by CLIA) were non-normally distributed variables, they were transformed with natural logarithm before correlation analysis. Student T-test was used to compare variables with a normal distribution, and Wilcoxon rank sum test was used for nonparametric variables between two groups. Categorical variables were compared by *χ*^2^ analysis. Spearman's rank correlation test and linear regression were performed to compare correlation between two factors. Bland–Altman plots and receiver operator characteristics (ROC) curve analysis were used to determine the diagnostic accuracy. Area under the curve (AUC) was employed to compare the diagnostic accuracy between ARR and ADRR. Youden index was used to determine the optimal cutoff value with corresponding sensitivity and specificity. The significance was set at P* *<* *0.05.

## 3. Results

### 3.1. Demographic and Clinical Characteristics of Patients

Between March 2016 and July 2018, 450 hypertensive patients with suspected PA were screened in our research center. Of them 386 patients had PH, and 64 had PA (14.2%). The demographic and clinical characteristics of the patients included in this study are shown in Table 1. In general, the enrolled patients were middle aged and overweight; sodium intake and renal function were normal (Table 1). As expected, lower serum K^+^ accompanied by higher urinary K^+^, lower PRA/DRC, and higher PAC (Table 3) was evidenced in PA patients.

### 3.2. Relationship between PRA and DRC

**Figure 2:**
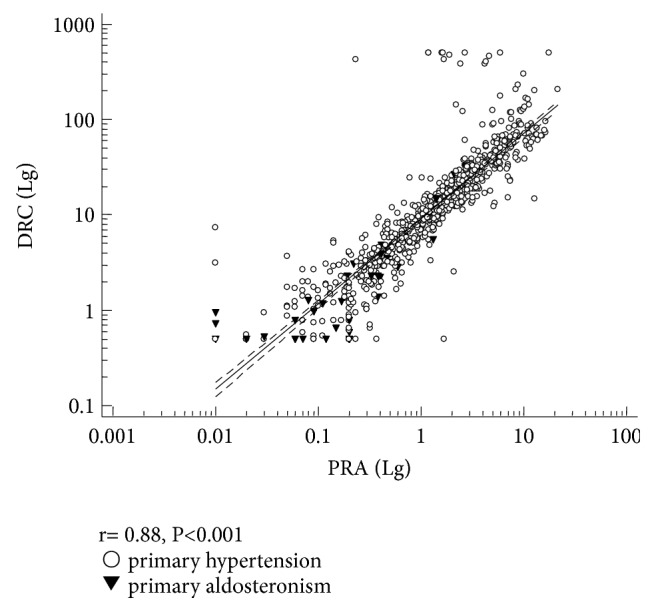
*Regression curve of PRA and DRC.* Dashed lines: confidence interval; continuous line: regression curve. R^2^=0.7662, Y=0.9573+0.8925×X. PRA: plasma renin activity; DRC: direct renin concentration.

To evaluate the within-patient correlation between PRA and DRC, we compared PRA measured by RIA with DRC measured by CLIA in both screening and confirmatory tests. The values of PRA and DRC in screening and confirmatory tests were shown in Table 3. PRA showed a significant within-patient correlation with DRC (r=0.88, P<0.001). To obtain a normal distribution, the values of PRA and DRC were converted to the natural logarithms. After that, we established the linear regression formula: R^2^=0.7662, Y=0.9573+0.8925×X (Figure 2). We also assessed the correlation between PRA and DRC when PRA was less than 1 ng/ml/h. The correlation was low but still statistically significant (r=0.12, P<0.001), and linear regression formula was R^2^=0.01612, Y=0.9512+0.1714×X. Our results suggested that there was a significant between-method correlation for all ranges of PRA/DRC.

### 3.3. Relationship between PAC Measured by RIA and PAC Measured by CLIA

**Figure 3:**
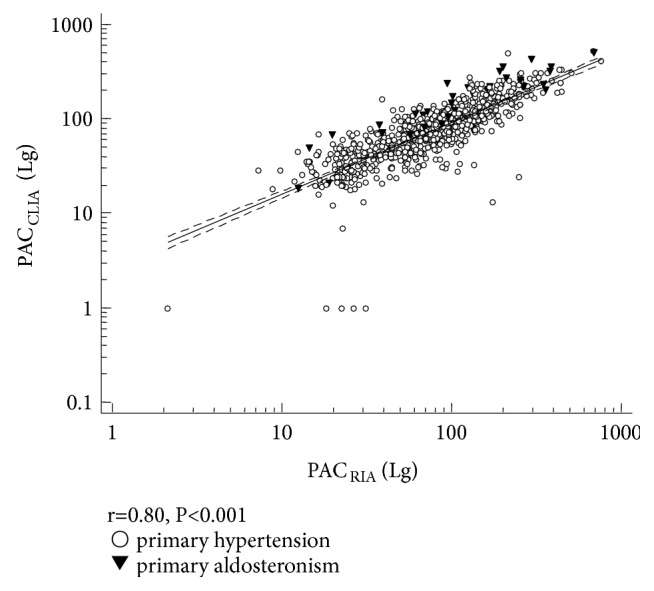
*Regression curve of PAC*_*RIA*_ *and PAC*_*CLIA*_. Dashed lines: confidence interval; continuous line: regression curve. R^2^=0.6439, Y=0.4431+0.7562×X. PAC_RIA_: plasma aldosterone concentration measured by radioimmunoassay; PAC_CLIA_: plasma aldosterone concentration measured by chemiluminescent immunoassay.

The values of PAC_RIA_ and PAC_CLIA_ in screening and confirmatory tests were shown in Table 3. Similarly, both screening and confirmatory tests values showed good correlation between PAC_RIA_ and PAC_CLIA_ (r=0.80, P<0.001). The linear regression (R^2^=0.6439, Y=0.4431+0.7562×X) was shown in Figure 3. These results suggested that there was a significant correlation between PAC derived from two assays methods.

### 3.4. Diagnostic Accuracy of ARR and ADRR

**Figure 4:**
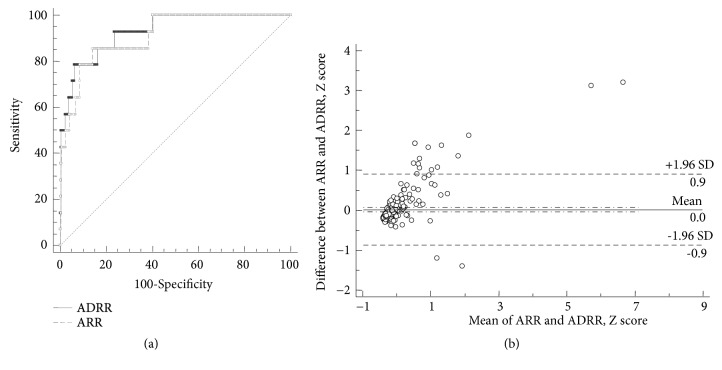
*ROC curves and Bland–Altman plot for ADRR and ARR.* (a) This analysis was performed based on the screening test values of ADRR and ARR. Continuous line: ROC curve for ADRR, 0.929 (95% CI: 0.894-0.955); dashed line: ROC curve for ARR, 0.910 (95% CI: 0.873-0.940). P=0.42. (b) There was no significant systematic bias. ADRR: aldosterone-to-direct renin concentration ratio; ARR: aldosterone-to-plasma renin activity ratio; CI: confidence interval.

ARR and ADRR were calculated by the equations as described above. To assess the diagnostic accuracy of ARR and ADRR, ROC curve analysis was performed (Figure 4(a)). The AUC of ARR was 0.910 [95% confidence interval (CI): 0.873-0.940], and 0.929 (95% CI: 0.894-0.955) for ADRR, respectively (P=0.42). Furthermore, a Bland–Altman plot of ARR and ADRR was generated. Because of different units of these two values, Z scores were used to avoid creating artificial proportional error. As shown in Figure 4(b), there was no significant systematic bias and only 15 out of 450 values (3%) fell out of the 95% CI. Our analysis suggested that there was a very good agreement between ADRR and ARR values.

### 3.5. Optimal Cutoff Value of ADRR for Identification of PA

In order to determine the optimal cutoff value of ADRR for identification of PA, we calculated the maximum Youden index. It revealed that the optimal cutoff value of ADRR was 2.93 (ng/dL)/(mU/L), sensitivity 80.33%, and specificity 92.11%, Table 4. The optimal cutoff value for ARR was 25.28 (ng/dL)/(ng/mL/h), sensitivity 76.92%, and specificity 93.38%, Table 5. Satisfactory specificity and sensitivity values were achieved by both methods. Our results thus suggest that ADRR with the cutoff value of 2.93 (ng/dL)/(mU/L) is suitable to be used as a screening index for PA with satisfying sensitivity and specificity.

## 4. Discussion

In this study, we analyzed clinical data from a relative large PA cohort (n=64). We investigated the diagnostic accuracy and optimal cutoff of ADRR for screening PA based on data from 450 hypertensive patients. We also estimated the between-method concordance of RIA and CLIA for examination PRA/DRA and PAC. Our results thus suggest that ADRR with the cutoff value of 2.93 (ng/dL)/(mU/L) is suitable to be used as a screening index for PA with satisfying sensitivity and specificity. The present study thus provides new evidence for a raw cutoff value of ADRR in screening test of PA.

In the light of the higher cardiovascular morbidity and mortality of PA patients [4, 15], it is essential to explore a feasible, sensitive, reproductive, and time-saving method useful for the early diagnose of PA for the purpose of timely decision making regarding the suitable targeted therapy options. During these years, ARR is recommended by the Endocrine Society guidelines as the reliable screening test for PA [4, 8]. Currently, DRC derived by CLIA measurement is emerging and more and more used in the daily practice [1]. Compared with traditional RIA measurement, CLIA measurement has plenty of advantages, including independence on renin substrate availability; being simple, sensitive, and pollution-free; and saving time and human resources [13].

In our study, we enrolled a large sample of 450 patients with suspected PA. All enrolled patients in our study underwent screening test and confirmatory test (saline infusion test) and were determined according to the Endocrine Society guidelines [4]. The patients with indeterminate diagnosis underwent additional confirmatory test (captopril challenge test) to reach an ultimate diagnosis. PA was diagnosed by RIA at confirmatory test in the present study. Finally, 64 patients were diagnosed as PA and the prevalence was 14.2% among patients with suspected PA, which was similar to that reported by previous studies [2, 16]. The results indicate the importance of screening PA among hypertensive patients with suspected PA.

According to Endocrine Society guidelines published in 2016 [4], ARR was still recommended as the method of choice for screening PA, despite the emerging evidence obtained with ADRR. The reason for this might be that the accurate cutoff value of ADRR is still controversial [4, 17, 18]. Therefore, ADRR has not been widely used in daily clinical practice [1]. In the present study, we assessed the diagnostic accuracy and cutoff value of ADRR as screening test of PA, with ARR serving as the reference method. We verified a significant correlation for all ranges of PRA/DRC and PAC_RIA_ with PAC_CLIA_. Regretfully, there was low correlation between PRA and DRC when PRA was less than 1 ng/ml/h. Relative low sensitivity of RIA and a lot of other factors that might affect the accuracy of this test, such as temperature and concentration of angiotensinogen in plasma, might be responsible for this finding. In addition, diagnostic accuracy of ADRR and ARR was comparable by ROC curve and Bland–Altman plot. Results were similar to previous reports [14, 19], in that ADRR derived from by CLIA measurement is suitable for screening PA. In the present study, the results revealed that the optimal cutoff value of ADRR was 2.93 (ng/dL)/(mU/L) with the corresponding sensitivity of 80.33% and specificity of 92.11%; for the ARR, the optimal cutoff value was 25.28 (ng/dL)/(ng/mL/h), and the sensitivity and specificity were 76.92% and 93.38%, respectively. Satisfactory specificity and sensitivity were achieved by both methods. The cutoff values obtained from previous studies were 2.06 (ng/dL)/(mU/L), 3.7 (ng/dL)/(mU/L), and 2.0 (ng/dL)/(mU/L), respectively [13, 14, 20]. The cutoff value defined in our study, 2.93 (ng/dL)/(mU/L), was close to the average value of the previous results. Compared with previous studies, patients in our study underwent complete prescreening preparation examinations, including pharmacological wash-out, correction of serum potassium, and dietary preparation, which ensures the quality control of the study with reasonable cutoff values.

There are some potential limitations in our study. Although a large sample of patients is collected to achieve acceptable results, the present study is just a single center research. Multicenter research is urgently needed to obtain a more reliable and potent cutoff of ADRR in screening PA, which we will investigate in the future study.

## 5. Conclusions

In conclusion, we demonstrated an optimal cutoff value of ADRR for screening PA with satisfactory sensitivity and specificity. We also confirmed the significant between-method correlation between RIA and CLIA derived PRA/DRC and PAC values. Our results might be of clinical importance for accelerating the clinical use of ADRR in screening PA. Future studies are warranted to validate the cutoff value derived from this study and from previously suggested cutoff values to establish the general cutoff value for optimal screening of PA patients.

## Figures and Tables

**Table 1:** Baseline characteristics of included hypertensive patients.

Variable	All patients (n=450)
Age (years)	48 ± 12
Sex (M/F)	251/199
BMI (kg/m^2^)	26.44 ± 3.89
SBP (mmHg)	146 ± 16
DBP (mmHg)	94 ± 11
HR (beat/minute)	72 ± 9
Serum K^+^(mmol/L)	3.65 ± 0.40
Serum Na^+^(mmol/L)	142.64 ± 3.09
Urinary K^+^(mmol/24h)	43.98 ± 19.81
Urinary Na^+^(mmol/24h)	169.82 ± 85.11
Serum creatinine (*μ*mol/L)	70.20 ± 19.64

PH: primary hypertension; PA: primary aldosteronism; BMI: body mass index; SBP: systolic blood pressure; DBP: diastolic blood pressure; HR: heart rate; K^+^: potassium; Na^+^: sodium. Age, BMI, SBP, DBP, HR, serum K^+^/Na^+^, urinary K^+^/Na^+^, and serum creatinine are expressed as mean ± standard deviation.

**Table 2:** Detection ranges and sensitivities of angiotensin I, DRC, and PAC measured by RIA and CLIA.

Variable	Detection range	Sensitivity
Angiotensin I (ng/ml)	0.20-12.00	≤0.10
PAC_RIA_ (ng/dL)	0.76-160.00	0.76
DRC (mU/L)	0.52-500.00	0.13
PAC_CLIA_ (ng/dL)	3.00-100.00	≤0.97

Plasma renin activity measured by radioimmunoassay was indirectly estimated by efficiency of generating angiotensin I from angiotensinogen. RIA: radioimmunoassay; CLIA: chemiluminescent immunoassay; PAC_RIA_: plasma aldosterone concentration measured by radioimmunoassay; DRC: direct renin concentration; PAC_CLIA_: plasma aldosterone concentration measured by chemiluminescent immunoassay.

**Table 3:** Anthropometric and clinical characteristics of the hypertensive patients screened in our study.

Variable	PH	PA	P value
	(n=386)	(n=64)	PH vs. PA
Age (years)	48 ± 12	49 ± 10	0.557
Sex (M/F)	221/165	30/34	0.122
BMI (kg/m^2^)	26.51 ± 3.83	25.98 ± 4.31	0.358
SBP (mmHg)	146 ± 14	148 ± 23	0.649
DBP (mmHg)	94 ± 11	96 ± 10	0.103
HR (beat/minute)	72 ± 9	71 ± 6	0.115
Serum K^+^(mmol/L)	3.71 ± 0.38	3.31 ± 0.42	<0.0001
Serum Na^+^(mmol/L)	142.52 ± 3.16	143.34 ± 2.50	0.049
Urinary K^+^(mmol/24h)	41.56 ± 17.65	58.22 ± 25.34	<0.0001
Urinary Na^+^(mmol/24h)	171.77 ± 85.32	158.39 ± 83.73	0.273
Serum creatinine (*μ*mol/L)	70.28 ± 19.22	69.72 ± 22.17	0.832
PRA (recumbent) (ng/ml/h)	1.04 (0.43-2.26)	0.20 (0.13-0.26)	<* *0.0001
PRA (upright) (ng/ml/h)	2.46 (1.13-5.59)	0.41 (0.10-0.83)	<* *0.0001
PRA (post ivSLT)(ng/ml/h)	0.6 (0.25-1.59)	0.16 (0.02-0.25)	<* *0.0001
DRC (recumbent)(mU/L)	9.37 (3.61-19.68)	1.28 (0.54-2.72)	<* *0.0001
DRC (upright) (mU/L)	20.09 (10.01-49.72)	3.52 (1.34-7.48)	<* *0.0001
DRC (post ivSLT) (mU/L)	6.16 (2.07-14.03)	1.00 (0.50-2.24)	<* *0.0001
PAC_RIA_ (recumbent) (ng/dL)	7.84 (5.03-11.48)	11.90 (7.01-20.85)	<* *0.0001
PAC_RIA_ (upright) (ng/dL)	11.56 (7.84-16.73)	20.50(13.07-35.22)	<* *0.0001
PAC_RIA_ (post ivSLT) (ng/dL)	2.61 (2.13-3.62)	5.91 (1.96-9.50)	<* *0.0001
PAC_CLIA_ (recumbent) (ng/dL)	7.32 (5.21-10.40)	14.15(10.00,20.95)	<* *0.0001
PAC_CLIA_ (upright) (ng/dL)	10.70 (7.11-16.48)	21.70 (17.15-31.65)	<* *0.0001
PAC_CLIA_ (post ivSLT) (ng/dL)	3.89 (2.80-5.10)	9.80 (7.60-14.50)	<* *0.0001

PH: primary hypertension; PA: primary aldosteronism; BMI: body mass index; SBP: systolic blood pressure; DBP: diastolic blood pressure; HR: heart rate; K^+^: potassium; Na^+^: sodium; PRA: plasma renin activity; DRC: direct renin concentration; PAC_RIA_: plasma aldosterone concentration measured by radioimmunoassay; PAC_CLIA_: plasma aldosterone concentration measured by chemiluminescent immunoassay; ivSLT: intravenous saline loading test. Age, BMI, SBP, DBP, HR, serum K^+^/Na^+^, urinary K^+^/Na^+^, and serum creatinine are expressed as mean ± standard deviation; PRA, DRC, and PAC are expressed as median (25th–75th percentiles).

**Table 4:** Sensitivity, specificity, positive predictive value, and negative predictive value of different cutoff values of ADRR.

criterion	sensitivity	specificity	+PV	-PV
>1.58	88.52	78.95	31.8	98.4
>2.04	81.97	85.53	38.6	97.7
>2.33	80.33	88.16	43.0	97.6
**>2.93**	**80.33**	**92.11**	**53.1**	**97.7**
>3.52	70.49	92.54	51.2	96.6
>4.20	67.21	95.18	60.8	96.3
>4.91	65.57	96.06	64.9	96.2

ADRR: aldosterone-to-direct renin concentration ratio; +PV: positive predictive value; -PV: negative predictive value. ADRR is expressed in (ng/dL)/(mU/L). The cutoff value in bold was used in the present study.

**Table 5:** Sensitivity, specificity, positive predictive value, and negative predictive value of different cutoff values of ARR.

criterion	sensitivity	specificity	+PV	-PV
>6.14	92.31	60.98	20.8	98.6
>6.52	84.62	62.72	20.1	97.3
>16.07	76.92	87.80	41.2	97.2
**>25.28**	**76.92**	**93.38**	**56.4**	**97.3**
>27.05	61.54	93.38	50.8	95.6
>33.99	61.54	95.12	58.4	95.7
>40.22	46.15	97.91	71.0	94.2

ARR: aldosterone-to-plasma rennin activity ratio; +PV: positive predictive value; -PV: negative predictive value. ARR is expressed in (ng/dL)/(ng/mL/h). The cutoff value in bold was used in the present study.

## Data Availability

The data used to support the findings of this study are available from the corresponding author upon request.
